# AP-1/IRF-3 Targeted Anti-Inflammatory Activity of Andrographolide Isolated from *Andrographis paniculata*

**DOI:** 10.1155/2013/210736

**Published:** 2013-06-06

**Authors:** Ting Shen, Woo Seok Yang, Young-Su Yi, Gi-Ho Sung, Man Hee Rhee, Haryoung Poo, Mi-Yeon Kim, Kyung-Woon Kim, Jong Heon Kim, Jae Youl Cho

**Affiliations:** ^1^Department of Genetic Engineering, Sungkyunkwan University, Suwon 440-746, Republic of Korea; ^2^Department of Herbal Crop Research, National Institute of Horticultural & Herbal Science, Rural Development Administration, Suwon 441-707, Republic of Korea; ^3^College of Veterinary Medicine, Kyungpook National University, Daegu 702-701, Republic of Korea; ^4^Laboratory of Chemical Genomics, Korea Research Institute of Chemical Technology, Daejeon 305-600, Republic of Korea; ^5^School of Systems Biological Science, Soongsil University, Seoul 156-743, Republic of Korea; ^6^Animal Biotechnology Division, National Institute of Animal Science, RDA, Suwon 441-706, Republic of Korea; ^7^Cancer Cell and Molecular Biology Branch, Research Institute, National Cancer Center, Goyang, Gyeonggi 410-769, Republic of Korea

## Abstract

Andrographolide (AG) is an abundant component of plants of the genus *Andrographis * and has a number of beneficial properties including neuroprotective, anticancer, anti-inflammatory, and antidiabetic effects. Despite numerous pharmacological studies, the precise mechanism of AG is still ambiguous. Thus, in the present study, we investigated the molecular mechanisms of AG and its target proteins as they pertain to anti-inflammatory responses. AG suppressed the production of nitric oxide (NO) and prostaglandin E_2_ (PGE_2_), as well as the mRNA abundance of inducible NO synthase (iNOS), tumor necrosis factor-alpha (TNF-**α**), cyclooxygenase (COX)-2, and interferon-beta (IFN-**β**) in a dose-dependent manner in both lipopolysaccharide- (LPS-) activated RAW264.7 cells and peritoneal macrophages. AG also substantially ameliorated the symptoms of LPS-induced hepatitis and EtOH/HCl-induced gastritis in mice. Based on the results of luciferase reporter gene assays, kinase assays, and measurement of nuclear levels of transcription factors, the anti-inflammatory effects of AG were found to be clearly mediated by inhibition of both (1) extracellular signal-regulated kinase (ERK)/activator protein (AP)-1 and (2) I**κ**B kinase **ε** (IKK**ε**)/interferon regulatory factor (IRF)-3 pathways. In conclusion, we detected a novel molecular signaling pathway by which AG can suppress inflammatory responses. Thus, AG is a promising anti-inflammatory drug with two pharmacological targets.

## 1. Introduction

Acute and chronic inflammatory responses are both part of the natural defense mechanisms of the body's innate immune system, and through such reactions, the body is able to maintain immunological homeostasis. Numerous of reports continue to suggest that inflammatory responses are accompanied by serious diseases, including septic shock, cancer, atherosclerosis, rheumatoid arthritis, and diabetes [1, 2], implying that inflammatory responses are a critical patron to the pathophysiology of several diseases. Thus, manipulation of inflammatory responses may allow for prevention of serious diseases or symptoms. Indeed, significant effort has been focused on exploring the molecular mechanisms of inflammatory responses to find therapeutic targets and, in parallel, on developing strong and safe anti-inflammatory drugs to prevent or reverse many kinds of inflammation-related diseases. Inflammatory events trigger macrophage activation to produce nitric oxide (NO), prostaglandin E_2_ (PGE_2_), tumor necrosis factor (TNF)-*α* or interleukin (IL)-1*β* [3]. Accordingly, most studies have been performed using macrophages and various toll-like receptor (TLR) ligands such as lipopolysaccharides (LPSs).

Although macrophage-mediated inflammatory responses are not fully understood, several aspects have been characterized as follows: (1) interactions between surface receptors, such as TLR-4 or TLR-2, and their ligands are important [4]; (2) subsequent activation occurs between adapter-inducing interferon-*β* (TRIF) and adaptor molecules including myeloid differentiation response gene 88 (MyD88) and Toll/Interleukin-1 receptor domain containing; and (3) there is a significant cross-activation between MyD88, transforming-growth-factor *β*-activated kinase 1 (TAK1), and mitogen activated protein kinases (MAPKs) such as extracellular signal-regulated kinase (ERK), which is linked to activation of nuclear factor-*κ*B (NF-*κ*B) and activator protein (AP)-1 [4, 5] or TRIF, TANK-binding kinase (TBK), and I*κ*B kinase *ε* (IKK*ε*), which are linked to the stimulation of interferon regulatory factor (IRF)-3-mediated interferon (IFN)-*α*/*β* production [6].

Recent findings include the critical function of Janus kinase 2 (JAK2) and its counter-activated transcription factor—signal transducer and activator of transcription (STAT-1)—in regulating the expression of proinflammatory genes [7]. Although JAK2 activity linked to phosphorylation of STAT-1 or STAT-2 is increased by direct interaction with TLR4 on a time scale of a few minutes, a major mechanism of activation of this enzyme during inflammatory responses occurs during late phase autologous stimulation with type I IFN-*α*/*β*.

**Figure 1:**
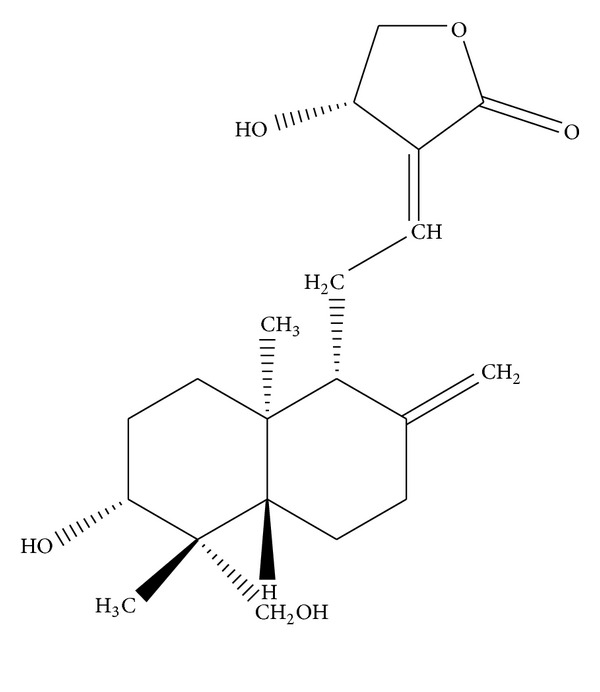
Chemical structure of AG.

AG (Figure 1) is a diterpenoid compound isolated from *Andrographis paniculata, *a medicinal plant ethnopharmacologically prescribed in Korea, China, and Japan [8]. AG exhibits antioxidative, anti-cancer, anti-inflammatory, antidiabetes, and antiaging properties [9, 10]. Although no AG derivatives have been tested in clinical trials to date, several investigators have tried to mass-produce AG and its derivatives due to its multiple inhibitory activities [11–14]. The proposed anti-inflammatory effects of AG are based on observations showing that it can suppress production of inflammatory mediators including NO, TNF-*α*, IL-6, IL-12, and PGE_2_ in activated macrophages [15, 16]. AG mediates its anti-inflammatory effects by inhibiting the activities of ERK1/2 (MAPK pathway) and AKT (PI3 K pathway) in LPS-induced macrophages [17]. In addition, the ability of AG to either activate protein phosphatase 2A and Akt or suppress Akt phosphorylation has been reported [18].

We performed a compound screen to identify anti-inflammatory drugs with high efficacy and low toxicity. Among several candidate compounds, AG was chosen for further study in macrophage-mediated inflammatory responses. Although several studies have reported that AG is an anti-inflammatory drug the molecular anti-inflammatory mechanisms of AG were still vague. Therefore, in this study, we focused on exploring novel molecular mechanisms of anti-inflammatory action of AG by studying TLR-mediated inflammatory responses in macrophages. Here, we perceive that AG exhibits anti-inflammatory effects *in vitro* and *in vivo* via novel pathways by inhibiting not only ERK/AP-1, but also IKK*ε*/IRF-3. 

## 2. Materials and Methods

### 2.1. Materials

AG (MW: 350.46; Figure 1), indomethacin, (3-4,5-dimethylthiazol-2-yl)-2,5-diphenyltetrazolium bromide, MTT, polyethylenimine (PEI), sodium carboxyl methylcellulose (Na CMC), pam3CSK, and LPS (*E. coli* 0111:B4) were purchased from Sigma-Aldrich Chemical Co. (St. Louis, MO, USA). Poly(I : C), U0126 (1,4-diamino-2,3-dicyano-1,4-bis[2-aminophenylthio] butadiene), SB203580 (4-[5-(4-fluorophenyl)-2-[4-(methylsulfonyl) phenyl]-1*H*-imidazol-4-yl]pyridine), SP600125 (1,9-pyrazoloanthrone), and AG490 (2-cyano-3-(3,4-dihydroxyphenyl)-N-(phenylmethyl)-2-propenamide) were purchased from Calbiochem (La Jolla, CA, USA). Rofecoxib (4-(4-methylsulfonylphenyl)-3-phenyl-5H-furan-2-one) was obtained from Amore Pacific R&D Center (Suwon, Korea). Lipofectamine 2000 was obtained from Invitrogen (Carlsbad, CA, USA). Luciferase constructs containing promoters for IRF-3 and AP-1 were a gift from Professor Chung, Hae Young (Pusan National University, Pusan, Korea), and Addgene (Cambridge, MA, USA). An enzyme immunoassay (EIA) kit for detecting PGE_2_ production was purchased from Amersham (Little Chalfont, Buckinghamshire, UK). The ERK kinase assay kit was purchased from Millipore (Billerica, MA, USA). Fetal bovine serum (FBS) and RPMI1640 were obtained from Gibco (Grand Island, NY, USA). RAW264.7 and human embryonic kidney 293 (HEK293) cells were purchased from ATCC (Rockville, MD, USA). All other chemicals were of analytical grade. Antibodies specific for either the total or phosphorylated forms of c-Fos, c-Jun, ATF2, CEBP, Fra-1, STAT-1, IRF-3, TBK1, IKK*ε*, tyrosine, threonine, serine, Janus kinase (JAK)2, extracellular signal-related kinase (ERK), c-Jun N-terminal kinase (JNK), p38, Akt, I*κ*B*α*, *γ*-tubulin, and *β*-actin were obtained from either Cell Signaling Technologies (Beverly, MA, USA) or Santa Cruz Biotechnology (Santa Cruz, CA, USA). 

### 2.2. Mice

Six-week-old male ICR, Balb/C, and C57BL/6 mice were purchased from Daehan Biolink (Chungbuk, Korea). Mice were given pellets (Samyang, Daejeon, Korea) and water *ad libitum* under a 12 h light/dark cycle. Studies were performed in accordance with guidelines established by the Kangwon National University Institutional Animal Care and Use Committee.

### 2.3. Preparation of Mouse Peritoneal Macrophages and Human Tonsil-Derived Macrophages

Peritoneal exudates were obtained from C57BL/6 male mice (7-8 weeks old, 17–21 g) by lavage 4 days after intraperitoneal injection of 1 mL of sterile 4% thioglycollate broth (Difco Laboratories, Detroit, MI, USA) as described previously [19]. After washing with RPMI 1640 medium containing 2% FBS, peritoneal macrophages (1 × 10^6^ cells/mL) were plated in 100 mm tissue culture dishes for 4 h at 37°C in a 5% CO_2_ humidified atmosphere. To prepare human tonsil-derived macrophages [20], surgically removed tonsils were cut into 2-3 mm thick pieces and digested for 15 min at 37°C with collagenase (Boehringer Mannheim, Germany). After rinsing with RPMI 1640 by centrifugation at 400 ×g for 7 min, cells were filtered through a 70 *μ*m nylon mesh. Next, red blood cells were depleted with Gey's solution, cells were washed with RPMI 1640 medium containing 2% FBS, and the resulting tonsil-derived cells were plated in Petri dishes. After four hours, adherent cells were collected using a cell scraper and used as macrophages. After washing the cells again, human tonsil-derived macrophages (2 × 10^6^ cells/mL) were plated to evaluate the PGE_2_ inhibitory activity of AG. The use of human tissues was approved by the Institutional Review Board of Yonsei University College of Medicine in Korea.

### 2.4. Cell Culture

RAW264.7 cells, a murine macrophage cell line, and HEK293 cells were maintained in RPMI 1640 or DMEM supplemented with 100 U/mL of penicillin, 100 *μ*g/mL of streptomycin, and 10% FBS. Cells were grown at 37°C and 5% CO_2_ in humidified air.

### 2.5. Determination of NO and PGE_2_ Production

RAW264.7 cells and peritoneal macrophages (1 × 10^6^ cells/mL) were preincubated for 18 h, after which cells were treated with AG (0 to 100 *μ*M) for 30 min and then further incubated with LPS (1 *μ*g/mL) for 24 h. NO production was determined by Griess assay and the production of PGE_2_ was determined by an enzyme immunoassay kit as described previously [21].

### 2.6. Cell Viability Assay

RAW264.7 cells (1 × 10^6^ cells/mL) were preincubated 18 h, after which AG (0 to 100 *μ*M) was added to the cell suspensions and incubated for 24 h. Cytotoxic effects of AG were evaluated by conventional MTT assay as described previously [22]. Briefly, 3 h prior to culture termination, 10 *μ*L of an MTT solution (10 mg/mL in phosphate buffered saline, pH 7.4) was added and cells were returned to the incubator. After 3 h, the MTT reaction was quenched by the addition of 15% sodium dodecyl sulfate into each well, which solubilized formazan produced by cells [23]. Absorbance at 570–630 nm (OD_570–630_) was measured with a Spectramax 250 microplate reader.

### 2.7. LPS-Induced Hepatitis Mouse Model

A model of experimental liver inflammation was induced by LPS injection according to a published method [24]. Briefly, fasted C57BL/6 mice were treated orally with AG (40 mg/kg) once a day for 6 days. One hour after the final administration of AG, LPS (10 mg/kg) or LPS (5 *μ*g/kg) and D-galactosamine (800 mg/kg) were administered intraperitoneally. Each animal was anesthetized with an overdose of urethane 1 h after administration of hepatitis inducers, and blood was drawn from the portal vein. The livers were then excised and gently rinsed under running tap water. Serum was obtained by centrifugation of blood at 3,000 rpm for 15 min. The levels of serum alanine aminotransferase (ALT) and aspartate aminotransferase (AST) were measured with a Roche Modular spectrophotometric autoanalyzer.

### 2.8. EtOH/HCl-Induced Gastritis Mouse Model

Experimental stomach inflammation was induced with EtOH/HCl according to a published method [25]. Fasted ICR mice were treated orally with AG (40 mg/kg) or ranitidine (40 mg/kg) twice per day for 3 days; the *in vivo* dosage of AG was determined based on previous *in vivo* tests in which 5 to 30 mg/kg was administered to mice [26]. Thirty minutes after the final injection of AG, 400 *μ*L of 60% ethanol in 150 mM HCl was administered orally. Each animal was anesthetized with an overdose of urethane 1 h after the administration of necrotizing agents. The stomachs were then excised and gently rinsed under running tap water. After opening the stomach along the greater curvature and spreading it out on a board, the area (mm^2^) of mucosal erosive lesions were measured using a pixel counter under blinded conditions.

### 2.9. mRNA Analysis by Semiquantitative Reverse Transcriptase (RT) and Real-Time Polymerase Chain Reaction

To determine the levels of mRNA expression of inflammatory mediators, total RNA was extracted from LPS-treated RAW264.7 cells with TRIzol Reagent (Gibco, Grand Island, NY, USA) according to the manufacturer's instructions. Total RNA was stored at −70°C until use. Semi-quantitative RT reactions were conducted as described previously [27, 28]. Quantification of mRNA was performed by real-time RT-PCR with SYBR Premix Ex Taq according to the manufacturer's instructions (Takara Bio, Inc., Shiga, Japan) using a real-time thermal cycler (Bio-Rad, Hercules, CA, USA) as described previously [29]. The primers used (Bioneer, Daejeon, Korea) are listed in Table 1.

### 2.10. Transfection and Luciferase Reporter Gene Assays

HEK293 or RAW264.7 cells (1 × 10^6^ cells/mL) were transfected with 1 *μ*g of plasmids containing TRIF, TBK1, AP-1-Luc, IRF-3-Luc, iNOS-Luc, COX-2-Luc, and *β*-galactosidase in the absence or presence of PMA/ionomycin or LPS using PEI or lipofectamine 2000 in a 12-well plate as described previously [30] or according to manufacturer's instruction. Cells were incubated for 48 h after transfection and then used for assays. Luciferase assays were performed using the Luciferase Assay System (Promega, Madison, WI, USA) as previously reported [31].

### 2.11. Preparation of Cell Lysates, Nuclear Fractionation, and Analysis by Immunoblotting and Immunoprecipitation

Livers from LPS-treated mice, RAW264.7 cells, or HEK293 cells (5 × 10^6^ cells/mL) were washed 3 times in cold PBS with 1 mM sodium orthovanadate and lysed in lysis buffer (20 mM Tris-HCl, pH 7.4, 2 mM EDTA, 2 mM ethyleneglycotetraacetic acid, 50 mM *β*-glycerophosphate, 1 mM sodium orthovanadate, 1 mM dithiothreitol, 1% Triton X-100, 10% glycerol, 10 *μ*g/mL aprotinin, 10 *μ*g/mL pepstatin, 1 mM benzimide, and 2 mM PMSF) for 30 min under rotation at 4°C. Lysates were clarified by centrifugation at 16,000 ×g for 10 min at 4°C and stored at −20°C until use. Nuclear lysates were prepared in a three-step procedure [32]. After treatment, cells were first collected with a rubber policeman, washed with 1× PBS, and lysed in 500 *μ*L of lysis buffer (50 mM KCl, 0.5% Nonidet P-40, 25 mM HEPES (pH 7.8), 1 mM phenylmethylsulfonyl fluoride, 10 *μ*g/mL leupeptin, 20 *μ*g/mL aprotinin, and 100 *μ*M 1,4-dithiothreitol (DTT)) on ice for 4 min. Next, cell lysates were centrifuged at 19,326 ×g for 1 min in a microcentrifuge. In the second step, the pellet (nuclear fraction) was washed once with wash buffer, which had the same composition as lysis buffer lacking Nonidet P-40. In the final step, nuclei were treated with an extraction buffer containing 500 mM KCl, 10% glycerol, and several other reagents mentioned above for the lysis buffer. The nuclei/extraction buffer mixture was frozen at −80°C, and then thawed on ice and centrifuged at 19,326 ×g for 5 min. The resulting supernatant was collected as the nuclear extract. Soluble cell lysates were immunoblotted and protein levels were quantified as described previously [33]. For immunoprecipitation, cell lysates containing equal amounts of protein (500 *μ*g) from RAW264.7 cells (1 × 10^7^ cells/mL) were precleared with 10 *μ*L of protein A-coupled Sepharose beads (50% v/v) (Amersham, Little Chalfont, Buckinghamshire, UK) for 1 h at 4°C. Precleared samples were then incubated with 5 *μ*L of antibody for IKK*ε*, IRF-3, phospho-ERK, or TBK1 overnight at 4°C. Immune complexes were mixed with 10 *μ*L of protein A-coupled Sepharose beads (50% v/v) and incubated for 3 h at 4°C.

### 2.12. ERK and IKK*ε* Enzyme Assay

To determine the inhibitory effects of AG or U0126 on LPS-activated ERK or IKK*ε* enzyme activities, immunoprecipitated phospho-ERK or IKK*ε* prepared from RAW264.7 cells (5 × 10^6^ cells/mL) treated with LPS (1 *μ*g/mL) for 30 (ERK) or 10 (IKK*ε*) min were incubated with AG or U0126 in the presence or absence of substrate protein [myelin basic protein (MBP)] or immunoprecipitated IRF-3 from unstimulated RAW264.7 cells, according to the manufacturer's instructions. The ERK or IKK*ε* kinase activities were determined with anti-phospho-MBP or anti-phospho-IRF-3 antibodies after immunoblotting analysis.

### 2.13. Statistical Analysis

Data are presented as the mean ± SD of experiments performed with 6 biological replicates (*n* = 6) per treatment and subjected to statistical analysis. Similar experimental data were observed in three independent experiments. For statistical comparisons, results were analyzed using ANOVA with Scheffe's post hoc test or Kruskal-Wallis/Mann-Whitney test. Differences with values of *P* < 0.05 were considered statistically significant. All statistical tests performed using SPSS (SPSS Inc., Chicago, IL).

## 3. Results

### 3.1. AG Suppresses the Production of Inflammatory Mediators in Macrophages

**Figure 2:**
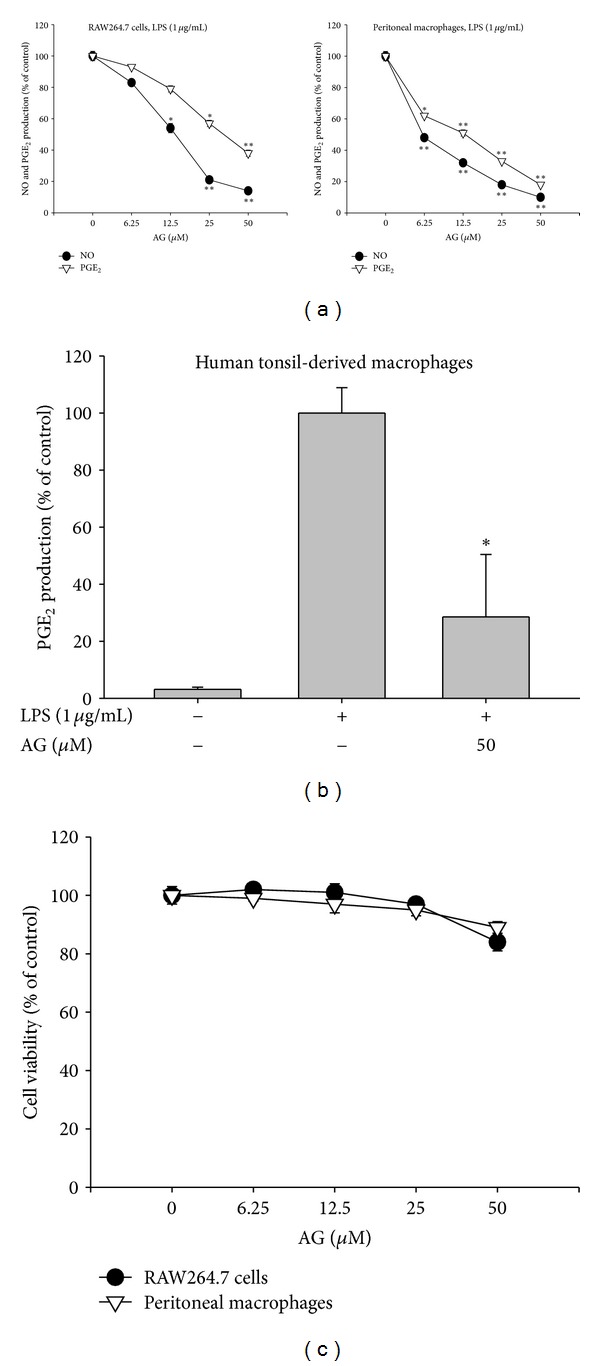
The effect of AG on production of inflammatory mediators and cell viability: (a) RAW264.7 cells (1 × 10^6^ cells/mL) (left panels), peritoneal macrophages (2 × 10^6^ cells/mL) (right panels) or (b) human tonsil-derived macrophages (2 × 10^6^ cells/mL) were treated with AG in the absence or presence of LPS (1 *μ*g/mL) for 24 h. Supernatants were collected and the concentration of NO and PGE_2_ in the supernatant was determined by Griess assay and EIA as described in Section 2. (c) Viability of RAW264.7 cells (1 × 10^6^ cells/mL) and peritoneal macrophages (2 × 10^6^ cells/mL) treated with AG were evaluated by MTT assay. Data are presented as the mean ± SD of an experiment done with 6 biological replicates (*n* = 6) per treatment. *P < 0.05 and **P < 0.01 compared to control group.

AG inhibited release of NO and PGE_2_ in macrophages activated by different TLR stimulators, including LPS, poly(I : C), and pam3CSK. AG suppressed LPS-induced production of NO and PGE_2_ in both RAW264.7 cells and peritoneal macrophages, which had a purity of 98.3% as determined by flow cytometry (data not shown), in a dose-dependent manner (Figure 2(a)) with IC_50_ values ranging from 6.4 to 36.7 *μ*M (Table 2). AG also suppressed the production of NO and PGE_2_ in RAW264.7 cells and peritoneal macrophages activated by poly(I : C) or pam3CSK in a dose-dependent manner, with IC_50_ values ranging from 13.1 to 39.5 *μ*M and from 16.3 *μ*M to 43.7 *μ*M, respectively (Table 2). Moreover, PGE_2_ production in LPS-treated human tonsil-derived macrophages was also remarkably suppressed upon treatment with AG (50 *μ*M) (Figure 2(b)). The viability of RAW264.7 cells and peritoneal macrophages was not affected by exposure to AG for either 6 or 12 h, and treatment for 24 h produced only weak cytotoxicity (less than 20%) at the highest AG concentration tested (50 *μ*M) (Figure 2(c)). Similarly, the AG derivative dehydro-AG also strongly inhibited NO production in LPS-treated RAW264.7 cells at 50 *μ*M with no cytotoxicity (data not shown).

### 3.2. AG Suppresses the mRNA Expression of Inflammatory Genes in Macrophages

**Figure 3:**
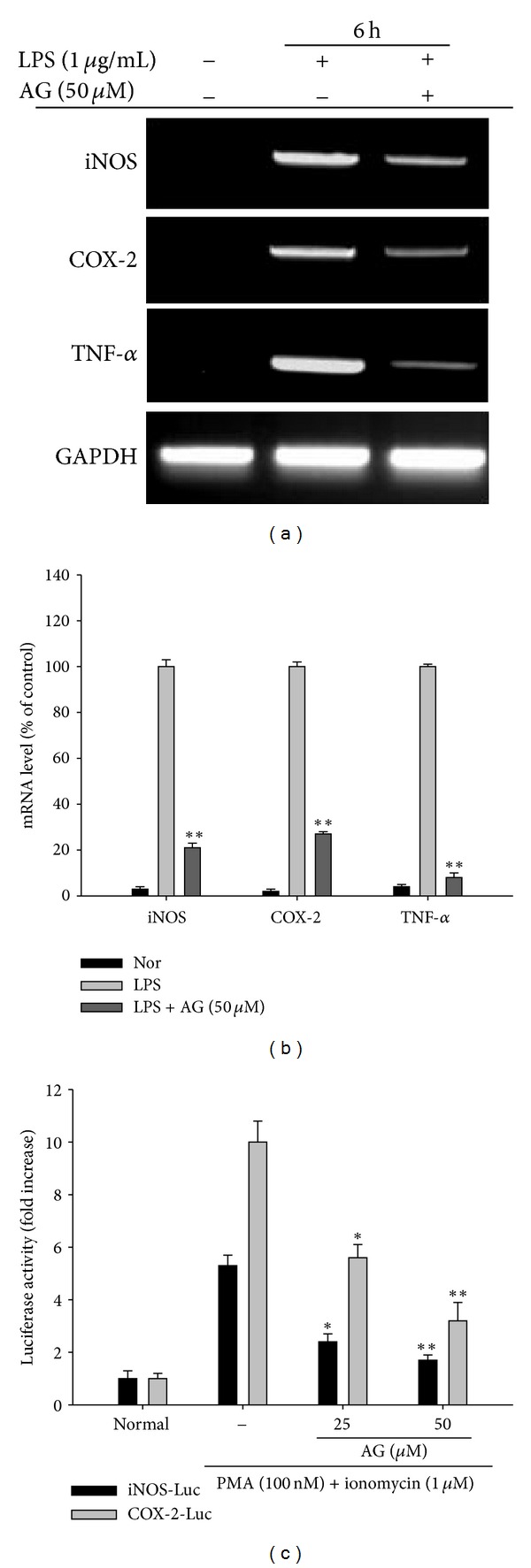
The effect of AG on mRNA expression of iNOS, COX-2, and TNF-*α* genes in LPS-treated RAW264.7 cells. RAW264.7 cells (5 × 10^6^ cells/mL) were incubated with AG in the absence or presence of LPS (1 *μ*g/mL) for 6 h. mRNA levels for iNOS, COX-2, and TNF-*α* were determined by (a) semi-quantitative PCR and (b) by real-time PCR. GAPDH was used as an internal control. (c) The promoter activities of iNOS and COX-2 were determined by luciferase reporter gene assay in HEK293 cells as described in Section 2. Data ((b) and (c)) are presented as the mean ± SD of an experiment done with 6 biological replicates (*n* = 6) per treatment. (a) is a representative image of three different gels with similar results. *P < 0.05 and **P < 0.01 compared to control group.

Semi-quantitative RT-PCR (Figure 3(a)) and real-time PCR (Figure 3(b)) revealed that AG (50 *μ*M) strongly suppressed the mRNA expression of inflammatory genes (iNOS, COX-2, and TNF-*α*) in RAW264.7 cells. To confirm the suppressive effects of AG towards these inflammatory mediators at the transcriptional level, reporter gene assays were conducted using constructs containing either the COX-2 or iNOS promoter region linked to a luciferase gene. In accordance with our PCR results, AG strongly suppressed PMA/ionomycin enhanced promoter activity of iNOS and COX-2 in a dose-dependent manner (Figure 3(c)).

### 3.3. AG Inhibits Activation of AP-1 and STAT-1 in Macrophages

**Figure 4:**
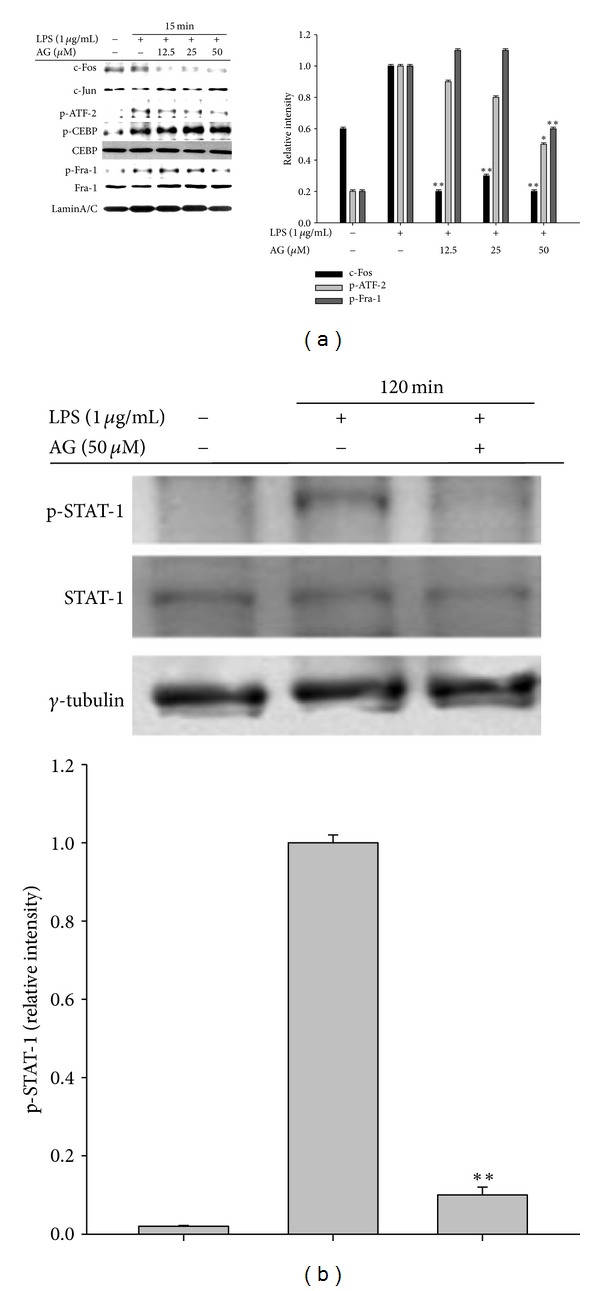
The effect of AG on nuclear translocation of total or phosphoforms of transcription factors. ((a), (b)) RAW264.7 cells (5 × 10^6^ cells/mL) were incubated with AG in the absence or presence of LPS (1 *μ*g/mL) for the indicated time. After preparing nuclear fractions, levels of total or phospho-forms of nuclear localized transcription factors, namely, (a) c-Fos, c-Jun, p-ATF-2, p-CEBP, p-Fra-1, CEBP, and Fra-1 and (b) p-STAT-1 and STAT-1 were identified by immunoblotting. Relative intensities were calculated with a DNR Bioimaging system (GelQuant software version. 2.7). Data ((a) right panel and (b) lower panel) are presented as the mean ± SD of relative intensity values of 3 blots (*n* = 3). *P < 0.05 and **P < 0.01 compared to control group.

The nuclear translocation patterns of p65 (NF-*κ*B), members of the AP-1 family (c-Fos, c-Jun, ATF-2, Fra-1, and CEBP), and STAT-1 are shown in Figures 4(a) and 4(b), and AG suppressed nuclear localization of c-Fos, phospho-ATF-2, and phospho-STAT-1. However, unlike previous reports [34], AG did not block the LPS stimulated increase in nuclear translocation of p65 levels after 15 min (data not shown). Together, these findings suggest that upstream signaling events for activation of the AP-1 family and STAT-1 are major targets of AG in LPS-mediated inflammatory responses.

### 3.4. ERK is a Primary Target of AG in Inhibition of AP-1 Activation

**Figure 5:**
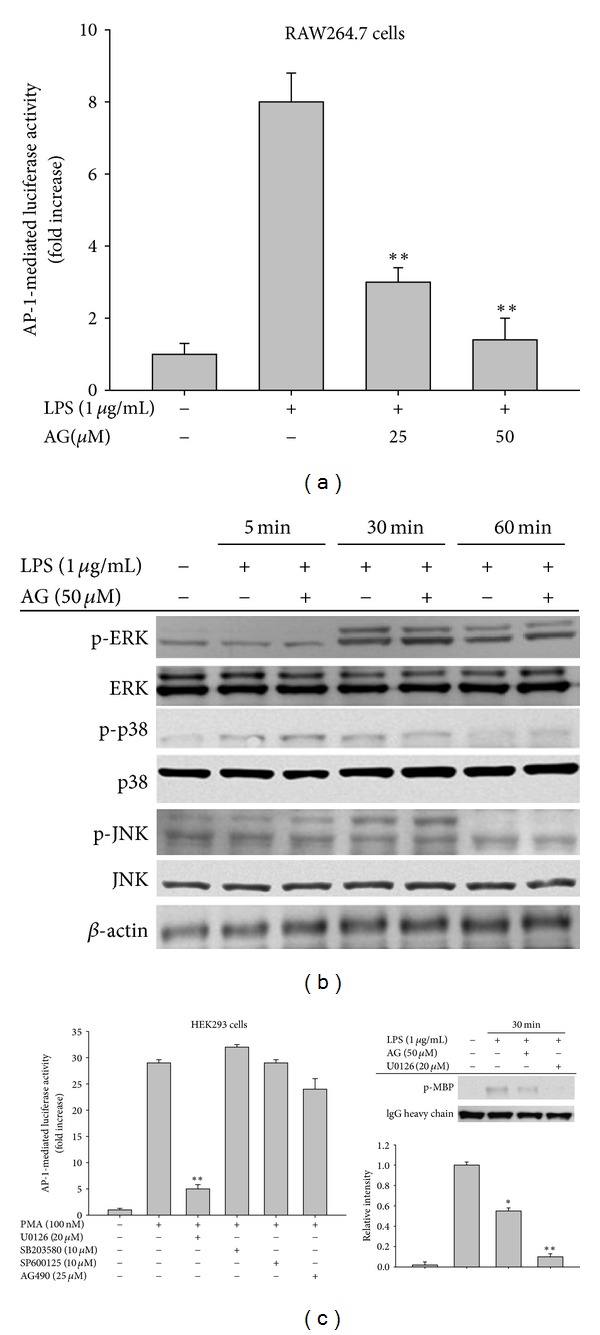
The effect of AG on AP-1 activation. (a) RAW264.7 cells cotransfected with AP-1-luciferase and *β*-gal constructs (as a transfection control) were treated with AG in the absence or presence of LPS (1 *μ*g/mL) for 12 h. Luciferase activity was determined by luminescence as described in Section 2. (b) RAW264.7 cells (5 × 10^6^ cells/mL) were incubated with AG in the absence or presence of LPS (1 *μ*g/mL) for the indicated times. After preparing whole cell lysates, levels of total or phospho-forms of upstream signaling enzymes (ERK, p38, and JNK) were identified by immunoblotting. ((c), left panel) HEK293 cells co-transfected with AP-1-luciferase and *β*-gal constructs were treated with MAPK inhibitors (U0126, SB203580, and SP600125) or the JAK2 inhibitor AG490 in the absence or presence of PMA (100 nM) for 12 h. Luciferase activities were determined by luminescence. ((c), right panel) A direct ERK enzyme assay was performed with immunoprecipitated phospho-ERK prepared from RAW264.7 cells (5 × 10^6^ cells/mL) incubated with LPS (1 *μ*g/mL) for 30 min in the presence or absence of AG or U0126 for 30 min, using an ERK kinase assay kit. Relative intensities of phospho-MBP level were calculated with a DNR Bioimaging system (Gelquant software version 2.7). Data are presented as the mean ± SD of an experiment done with 6 biological replicates (*n* = 6) per treatment ((a) and (c), left panels) or 3 different blots ((c), right lower panel). Other data (b) are representative images of three different experiments that had similar results. *P < 0.05 and **P < 0.01 compared to control group.

Reporter gene assays confirmed that AG significantly suppressed AP-1 activation triggered by LPS in RAW264.7 cells in a dose-dependent manner (Figure 5(a)). However, AG did not suppress NF-*κ*B activation induced by either TNF-*α* treatment or by cotransfection with MyD88, TRIF, or TBK1 (data not shown). This finding differs from that of previous reports [34]; however, we did find that TRAM-induced NF-*κ*B activation was diminished by AG in a dose-dependent manner (data not shown), suggesting that NF-*κ*B inhibition by AG appears to be signal dependent.

Because AP-1 translocation is mainly regulated by MAPKs, we investigated the possible involvement of ERK, p38, and JNK in AG inhibition. No inhibition of phospho-MAPK levels by AG was observed up to 60 min of incubation (Figure 5(b)), suggesting that AG does not inhibit the activities of upstream kinases responsible for MAPK phosphorylation. However, the possibility remained that AG could directly suppress the activities of ERK, p38, or JNK. Using a reporter gene assay, we next investigated the contribution of MAPK to AP-1 activation. As shown in Figure 5(c) (left panel), the ERK inhibitor U0126, but not other inhibitors, strongly attenuated AP-1-mediated luciferase activity, suggesting that ERK may be the major pathway involved in AP-1 translocation. To confirm whether AG is capable of abrogating the enhanced enzyme activity of ERK, a direct kinase assay was performed. As shown in Figure 5(c) (right panel), AG suppressed the phosphorylation of the ERK substrate MBP, although its inhibitory activity was not stronger than that of U0126. This result strongly suggests that AG is able to directly modulate the enzyme activity of ERK.

### 3.5. IKK*ε* is an Additional Target of AG in Inhibiting IRF-3 and STAT-1 Activation

**Figure 6:**
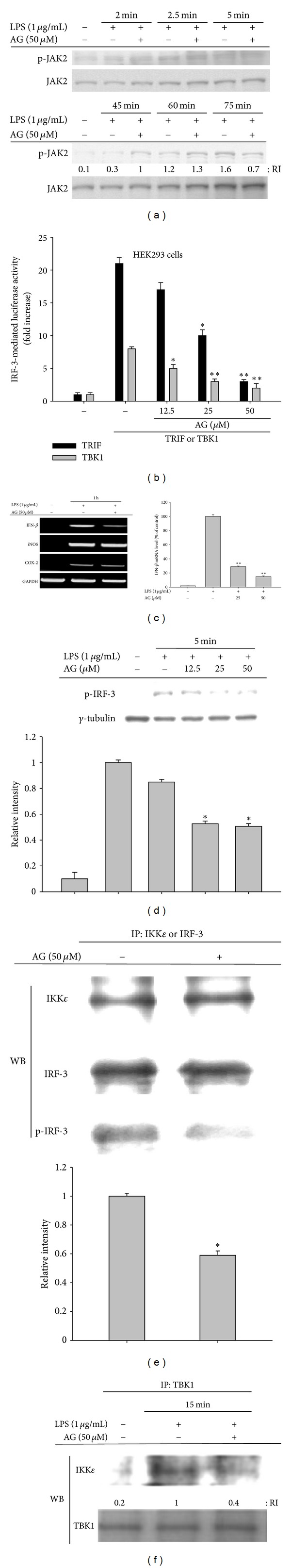
The effect of AG on JAK2/STAT-1 signaling. (a) RAW264.7 cells (5 × 10^6^ cells/mL) were incubated with AG in the absence or presence of LPS (1 *μ*g/mL) for the indicated times. After preparing whole cell lysates, levels of total or phospho-forms of JAK2 were examined by immunoblotting. (b) HEK293 cells cotransfected with IRF-3-luciferase and *β*-gal constructs (as a transfection control) were treated with AG in the absence or presence (cotransfection) of inducer protein constructs (TRIF or TBK1) for 12 h. Luciferase activity was determined by luminescence. (c) RAW264.7 cells (5 × 10^6^ cells/mL) were incubated with AG in the absence or presence of LPS (1 *μ*g/mL) for 1 h. Levels of mRNA for IFN-*β*, iNOS, and COX-2 were determined by semi-quantitative RT-PCR (left panel) or real-time PCR (right panel). (d) RAW264.7 cells (5 × 10^6^ cells/mL) were incubated with AG in absence or the presence of LPS (1 *μ*g/mL) for 5 min. After preparing nuclear fractions, levels of total or phospho-forms of IRF-3 were examined by immunoprecipitation and immunoblotting. (e) IKK*ε* kinase activity with AG was determined by a conventional kinase assay with immunoprecipitated IKK*ε* from RAW264.7 cells treated with LPS for 10 min and immunoprecipitated IRF-3 from normal RAW264.7 cells. (f) Molecular complex formation between TBK1 and IKK*ε* from LPS (1 *μ*g/mL)-treated RAW264.7 cells was examined by immunoblotting. Relative intensities were calculated with the DNR Bio-imaging system (Gelquant software version 2.7). Data are presented as the mean ± SD of an experiment done with 6 biological replicates (*n* = 6) per treatment (b), and (c) right panel) or 3 biological replicates (*n* = 3) per treatment ((d) lower panel and (e) lower panel). The other data ((a), (c) left panel, (d) upper panel, and (f)) are representative images of three different experiments that showed similar results. RI: Relative intensity. *P < 0.05 and **P < 0.01 compared to the control group.

Because the levels of phospho-STAT-1, but not total STAT-1, were decreased by AG in the nuclear fraction, we next examined the inhibitory mechanism of AG against STAT activation (Figure 4(b)). First, the possibility of JAK2 involvement, an essential requirement for the phosphorylation of STAT-1 [35], was tested. As shown in Figure 6(a), AG diminished the phosphorylation of JAK2 at 75 min up to 55%, but not at significantly earlier time points (2 to 5 min), suggesting that AG-mediated inhibition of JAK2 activation is not a direct/early event. Because late phase activation of JAK2 is known to be autologously activated by type I interferons such as IFN-*β*, we examined whether AG suppresses IFN-*β* production. As expected, AG clearly inhibited the upregulation of luciferase activity mediated by IRF-3 binding to the IFN-*β*-promoter region after cotransfection with TRIF or TBK1 (Figure 6(b)). Further, mRNA levels of IFN-*β* evaluated by RT-PCR (Figure 6(c) left panel) and real-time PCR (Figure 6(c) right panel) were decreased by AG. In agreement with these results, AG also suppressed nuclear levels of phospho-IRF-3 induced by LPS in a dose-dependent manner (Figure 6(d)). To examine whether AG inhibits the activities of upstream kinases, such as TBK1 and IKK*ε* [36, 37], which induce IRF-3 phosphorylation [36], we analyzed the activities of both TBK1 and IKK*ε* by *in vitro* kinase assay. Specifically, we looked at the formation of molecular complexes between these enzymes. As shown in Figure 6(e), while AG strongly blocked the kinase activity of IKK*ε* responsible for IRF-3 phosphorylation, the kinase activity of TBK1 was not blocked by AG (data not shown). Similarly, complex formation between IKK*ε* and TBK1, a critical step for the activation of IKK*ε*, was suppressed by AG up to 60% (Figure 6(f)). 

### 3.6. AG Ameliorates LPS-Induced Hepatitis and EtOH/HCl-Induced Gastritis in Animal Disease Models

**Figure 7:**
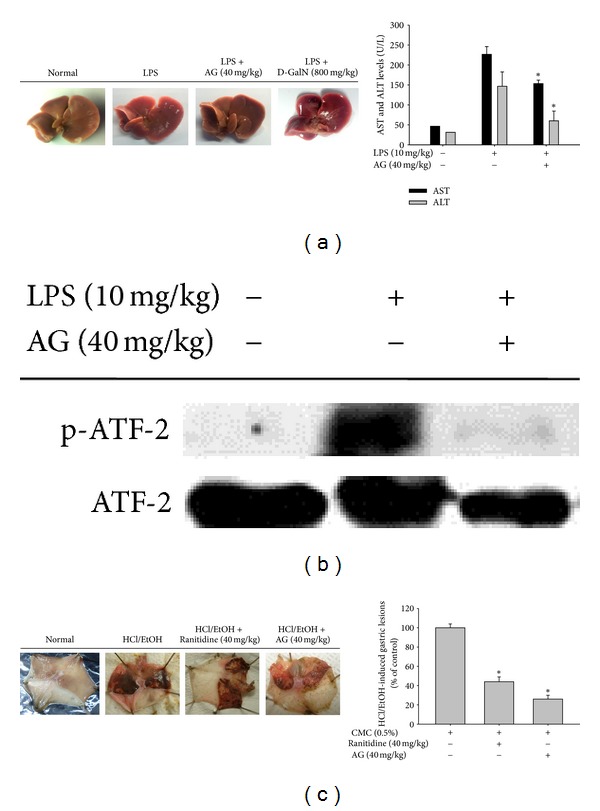
*In vivo* anti-inflammatory effect of AG on LPS-induced hepatitis and HCl/EtOH-induced gastritis animal models. Mice were administered AG orally for 6 days and treated intraperitoneally with LPS or LPS/D-galactosamine to induce hepatitis. After 1 h, ((a), left panel) livers were resected and photos were taken, ((a), right panel) after which serum was prepared to measure biochemical parameters (AST and ALT). (b) The inhibitory activity of AG on the phosphorylation of transcription factor ATF2 in the livers from LPS-treated mice was examined by immunoblotting. The levels of total or phospho-forms of ATF2 were measured from whole liver lysates prepared from LPS-treated mice. (c) Mice were orally administered with AG or ranitidine for 3 days and treated orally with EtOH/HCl to induce gastritis. After 1 h, ((c), left panel) stomachs were resected and photos were taken of the gastric lesions in the stomach. The areas of gastric lesions ((c), right panel) were measured. Data ((a) right panel and (c) right panel) are presented as the mean ± SD of an experiment done with 6 mice (*n* = 6). *P < 0.05 compared to the control group.

To examine whether AG has anti-inflammatory effects in animal models of inflammatory disease, we employed LPS-induced hepatitis and EtOH/HCl-induced gastritis in mice. Orally administered AG (40 mg/kg) significantly attenuated LPS-induced liver damage (Figure 7(a), right panel) and decreased elevated serum levels of ALT and AST derived from damaged hepatocytes (Figure 7(a), left panel). Conversely, *D*-galactosamine boosted LPS-induced hepatic damage, leading to a dramatic increase of serum parameters (ALT and AST) up to 1,527 and 1,950 U/L. We also examined whether AG could suppress enhanced phospho-ATF-2 level in livers from LPS-treated mice. In agreement with* in vitro *effect (Figure 4(a), left panel), AG strongly suppressed ATF-2 phosphorylation in the LPS-induced hepatitis mouse model (Figure 7(b)). In the case of the EtOH/HCl-induced gastritis model, AG strongly reduced the area of the gastric lesions similar to ranitidine (40 mg/kg) (Figure 7(c)) and rofecoxib (10 mg/kg), a selective COX-2 inhibitor (data not shown), suggesting that AG is an orally available anti-inflammatory drug with effects similar to those of known anti-inflammatory remedies [38].

## 4. Discussion

AG was originally identified as a component of the medicinal plant *Andrographis paniculata, *known in Korea as Cheonshimryeon and in China as Chuan Xin Lian, and has been traditionally prescribed for numerous inflammatory diseases including infection, inflammation, cold, fever, and diarrhea in Korea, China, and Japan [39, 40]. A significant body of scientific evidence supports the ethnopharmacological significance of *Andrographis paniculata* and its components with respect to their therapeutic efficacy against inflammatory diseases. Specifically, the ethyl acetate fraction of this plant strongly attenuates LPS-induced lung inflammation [41]. Likewise, carrageenan-induced inflammatory symptoms were also reported to be suppressed by methanolic extracts of *Andrographis paniculata *[42]. AG was identified as a major active component of *Andrographis paniculata* and has been demonstrated to have anti-inflammatory activity as assessed by the carrageenan-induced inflammation model [43], an ovalbumin-A-induced mouse asthma model [34] and an allergic-lung inflammation model [16]. Our experiments also clearly supported the anti-inflammatory effects of AG on inflammatory responses both *in vitro* and *in vivo*. AG suppressed the production of major inflammatory mediators such as NO and PGE_2_ in a dose-dependent manner, regardless of macrophage type (primary or cancer cells) (Figure 2) as well as inflammatory stimuli [LPS, pam3CSK, and poly(I:C)] (data not shown). The mild-conditioned hepatitis symptoms in LPS-treated mice (Figure 7(a)) as well as severe inflammatory stomach lesions in EtOH/HCl-treated mice (Figure 7(b)) were also robustly suppressed by orally administered AG (40 mg/kg).

 The anti-inflammatory effects of AG appear to be due to its ability to inhibit various inflammatory mediators at the transcriptional level. Our data indicated that AG-mediated inhibition was strongly linked to suppression of mRNA expression of inflammatory genes such as iNOS, COX-2, and TNF-*α* (Figure 3). In addition to our data, other research groups have shown that AG can strongly suppress transcriptional upregulation of proinflammatory cytokines (TNF-*α*, IL-1*β*, IL-4, IL-5, IL-6, and IL-13), cytokine receptors (IL-1*β*R and TNFR), adhesion molecules (E-selectin and VCAM-1), and chemokines (CCL8 and CXCL11) in macrophages, vascular endothelial cells, and eosinophils [34, 44, 45].

 In the present study, the COX-2 inhibitor rofecoxib strongly ameliorated HCl/EtOH-induced stomach inflammation (data not shown). Given that COX-1 inhibitors (aspirin, indomethacin, and other NSAIDs) are known to induce gastritis [46] and potent NOS inhibitors (e.g., NG-Nitro-L-arginine methyl ester) exacerbate the EtOH-induced gastric ulcer index by up to 2-fold [47], we considered the possibility that COX-2 activity or COX-2-mediated levels of PGE_2_ could play a pathophysiological role in the generation of EtOH/HCl-induced stomach inflammation. Thus, AG-mediated inhibition of COX-2 expression was considered to be one of the major contributing factors in its gastroprotective activity towards NO and TNF-*α*.

 The ability of AG to inhibit transcriptional activation has been mostly reported via its ability to suppress NF-*κ*B activation [48]. Regarding the inhibitory mechanism of AG, it has been demonstrated that AG binds to reduced cysteine 62 of the NF-*κ*B p50 subunit following thiolation, and AG fails to inhibit binding of p50 in a C62S mutant [48]. However, under our conditions, when NF-*κ*B activation was induced by LPS and TNF-*α* or cotransfection with adaptor molecules such as MyD88 and TRIF, we did not observe AG-mediated inhibition of p65 translocation and NF-*κ*B activation, although TRAM-induced NF-*κ*B activation in a reporter gene assay and LPS-induced I*κ*B*α* phosphorylation were both dose-dependently suppressed by AG (data not shown). On the contrary, there is a report that AG blocks p65 nuclear translocation and DNA-binding activity in nuclear extracts from lung tissues of OVA-challenged mice [34]. Likewise, a report that AG can increase protein phosphatase 2A activity and subsequent dephosphorylation of p65 at S536 [18] appears to support our results. According to our data, AP-1 and IRF-3 are critical transcription factor targets of AG pharmacology in LPS-treated macrophages. Specifically, AP-1-induced luciferase activity in LPS-activated RAW264.7 cells was suppressed by AG (Figure 5(a)), which also clearly decreased levels of both nuclear and whole cell c-Fos and phospho-ATF-2 in LPS-treated RAW264.7 cells (Figure 4(a)) and mouse livers (Figure 7(b)). Moreover, AG decreased IRF-3-mediated luciferase activity in HEK293 cells transfected with TRIF and TBK1 (Figure 6(b)) as well as the abundance of nuclear phospho-IRF-3 (Figure 6(d)). Together, these data suggest that the inflammatory pathways of the AP-1 family and IRF-3 could be major inhibitory targets of AG in TLR4-activated macrophages. Similar findings that support this possibility were previously observed in the downregulation of MMP-7 via the inactivation and decrease of nuclear AP-1 in A549 cells [49, 50] although there was no mention of IRF-3 as a target of AG action in these studies.

 In spite of numerous studies, the direct molecular targets of AG have not yet been fully elucidated. Previous studies have been limited mostly to simple identification of certain biochemical phenomena including the measurement of phosphorylated forms of enzymes found in apoptotic and inflammatory signaling cascades. For example, AG has been shown to reduce protein levels of activated Akt in an HRas-transformed rat kidney epithelial (RK3E) cell line [51], phosphorylation of ERK in the invasion of CT26 cells [52], enzyme activity of *α*-glucosidase [53], phosphorylation levels of ERK1/2 and AKT in LPS-treated macrophages [17], and phosphorylation of ERK1/5 induced by anti-CD3 antibody or PMA/ionomycin in T cells. However, none of these studies tested whether AG directly targets a specific kinase activity. Interestingly, ERK is an AP-1 upstream enzyme that was directly targeted by AG under our kinase assay conditions (Figure 5(c) right panel), unlike a previous report indicating that AG suppresses ERK phosphorylation [17, 54]. Nonetheless, our data and accumulated evidence in other reports strongly suggest that ERK is a primary target of AG pharmacology essential for the suppression of downstream transcription factor AP-1 with respect to both its anti-inflammatory and anti-cancer activities.

The JAK2/STAT-1 pathway inhibitor AG490 was previously shown to exhibit strong anti-inflammatory activity, and thus suppression of phospho-STAT-1 levels by AG is regarded as another critical event for its pharmacological action [55]. However, our data suggested that AG does not act as a direct inhibitor of JAK2/3, since early phosphorylation of the enzyme triggered by autophosphorylation [56] was not blocked by AG (Figure 6(a)) and JAK3 kinase activity was not reduced (data not shown). Rather, upstream signaling required for late phase JAK2/STAT-1 activation might be a potential target of AG. Interestingly, AG blocked early expression of IFN-*β* but not iNOS and COX-2 during LPS stimulation (Figure 6(c)) and reduced luciferase activity triggered by cotransfection of an IFN-*β* promoter-containing a reporter gene construct with IRF-3 binding sequences and TRIF or TBK1 (Figure 6(b)). Ultimately, our kinase assays led us to conclude that the kinase activity of IKK*ε*, but not TBK1, can be directly suppressed by AG (Figure 6(e)) and that molecular complex formation between TBK1 and IKK*ε* (Figure 6(f)) and phosphorylation of IRF-3 were subsequently inhibited (Figure 6(d)). Therefore, while our data strongly suggest that AG has dual independent inhibitory targets (ERK and IKK*ε*) in TLR-activated inflammatory signaling, further studies are needed to determine how AG can simultaneously inhibit two different enzymes, namely, ERK and IKK*ε*. Considering that AG is capable of binding to cysteine residues of target proteins (p50) [48], thiolation of specific cysteine residues in these enzymes, which is also seen in other chemically reactive compounds, such as benzene metabolites [57] and sesquiterpene lactones [21, 58], could be used to explain its biochemical mode of action. The possibility of AG-induced thiolation of both ERK and IKK*ε* will be verified in our next study.

## 5. Conclusions

**Figure 8:**
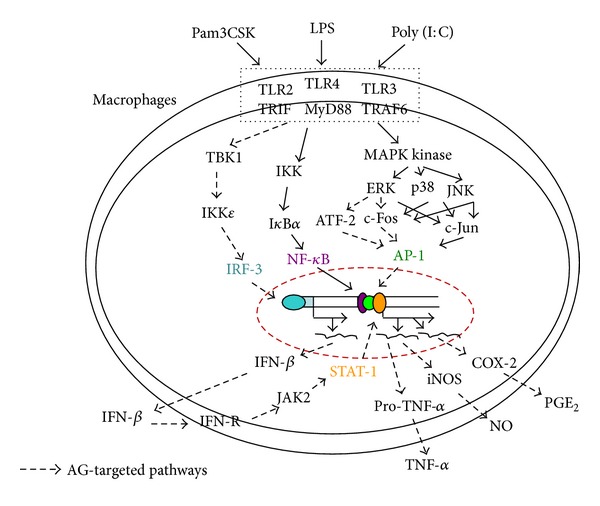
Schematic diagram of the proposed molecular anti-inflammatory mechanism of AG.

We demonstrated that AG strongly suppresses both *in vitro *and *in vivo *inflammatory responses in TLR-activated macrophages, such as the production of NO and PGE_2_, and the expression of proinflammatory genes, such as iNOS, COX-2, and TNF-*α*. AG also ameliorated the symptoms of both LPS-triggered hepatitis and EtOH/HCl-induced gastritis in mice. In particular, AG was shown to act as a dual inhibitor of ERK and IKK*ε*, which are particularly important in TLR4-mediated inflammation. As a consequence, AP-1 (c-fos/ATF-2) and IRF-3/JAK2/STAT-1 pathways were suppressed by AG, as summarized in Figure 8. AG-containing *Andrographis paniculata* has been ethnopharmacologically used for a long time and is orally available [59, 60] as well as easily detected in serum [61]. Furthermore, several carrier systems have been developed to improve the oral adsorption of AG [62], and chemical derivatization of AG has already been performed by many scientists [43, 63]. Thus, together with the existing literature, our data strongly suggest that AG and/or its derivatives could be used as novel anti-inflammatory agents. To support this possibility, we will use additional *in vivo *inflammatory models, such as acute (septic shock and carrageenan-induced arthritis) and chronic (collagen- or adjuvant-induced arthritis) disease conditions for future preclinical trials.

## Figures and Tables

**Table 1:** PCR primers used in this study.

Name		Sequence (5′ to 3′)
Real-time PCR		
iNOS	F	GGAGCCTTTAGACCTCAACAGA
	R	TGAACGAGGAGGGTGGTG
IFN-*β*	F	TCCAAGAAAGGACGAACATTCG
	R	GAGGCCATTTGGGAACTTCT
COX-2	F	CACTACATCCTGACCCACTT
	R	ATGCTCCTGCTTGAGTATGT
IRF3	F	GGCTGACTTTGGCATCTT
	R	TTCCTCTTCCAGGTTGACA
GAPDH	F	CAATGAATACGGCTACAGCAAC
	R	AGGGAGATGCTCAGTGTTGG
Semiquantitative PCR		
iNOS	F	CCCTTCCGAAGTTTCTGGCAGCAG
	R	GGCTGTCAGAGCCTCGTGGCTTTGG
TNF-*α*	F	TTGACCTCAGCGCTGAGTTG
	R	CCTGTAGCCCACGTCGTAGC
IFN-*β*	F	CAGGATGAGGACATGAGCACC
	R	CTCTGCAGACTCAAACTCCAC
COX-2	F	CACTACATCCTGACCCACTT
	R	ATGCTCCTGCTTGAGTATGT
GAPDH	F	CACTCACGGCAAATTCAACGGCA
	R	GACTCCACGACATACTCAGCAC

**Table 2:** Inhibitory potency of AG on the production of inflammatory mediators in RAW264.7 cells and peritoneal macrophages.

Cells	Ligand	IC_50_ value (*μ*M)	
		NO	PGE_2_
RAW264.7 cells	LPS	14.4 ± 0.3	36.7 ± 0.6
	Poly (I : C)	13.1 ± 0.6	28.9 ± 0.5
	Pam3CSK	16.3 ± 0.7	21.2 ± 0.5

Peritoneal macrophages	LPS	6.4 ± 0.8	11.3 ± 0.2
	Poly (I : C)	33.1 ± 1.3	39.5 ± 1.8
	Pam3CSK	17.4 ± 0.5	43.7 ± 1.7

RAW264.7 cells (1 × 10^6^ cells/mL) or peritoneal macrophages (2 × 10^6^ cells/mL) were treated with LPS (1 *μ*g/mL), poly(I : C) (200 *μ*g/mL), or pam3CSK (10 *μ*g/mL) in the presence or absence of AG for 24 h. Supernatants were collected and the concentrations of NO and PGE_2_ in the supernatants were determined by Griess assay and EIA, respectively, as described in Section 2.
